# Prevalence of precancerous gynecological lesions and gynecological cancer in patients undergoing vaginal hysterectomy for pelvic organ prolapse

**DOI:** 10.1080/07853890.2023.2273428

**Published:** 2023-10-26

**Authors:** Ornthicha Suphattanaporn, Apisith Saraluck, Jittima Mononai, Navamol Lekskul, Orawee Chinthakanan

**Affiliations:** Department of Obstetrics and Gynaecology, Faculty of Medicine Ramathibodi Hospital, Mahidol University, Bangkok, Thailand

**Keywords:** Pelvic organ prolapse, vaginal hysterectomy, precancerous gynecological lesion, gynecological cancer, preoperative evaluation

## Abstract

**Objectives:**

This retrospective cohort study aimed to determine the prevalence of precancerous or malignant lesions of the cervix and/or endometrium among patients who underwent vaginal hysterectomy.

**Materials and methods:**

Medical record of patients who had been diagnosed with pelvic organ prolapse (POP) and undergone vaginal hysterectomy from January 2009 to September 2018 in tertiary hospital was reviewed. The exclusion criteria included individuals who had previously received a diagnosis of gynecologic precancerous lesions or cancer, had abnormal preoperative findings, presented abnormal cervical cancer screening test results or abnormal results from preoperative endometrial biopsy, and had incomplete operative notes or pathological results.

**Results:**

The electronic medical records of 530 patients were reviewed and included in the analysis. Nine of the 530 patients (1.7%) had precancerous or malignant lesions of the cervix and/or endometrium. The prevalence of atypical endometrial hyperplasia and endometrial carcinoma were 0.19% and 0.57%, respectively. All of the patients with endometrial cancer subsequently underwent complete surgical staging. Precancerous cervical lesions were found in five patients (0.95%): CIN II 0.38% and CIN III 0.57%. No cases of cervical cancer were identified.

**Conclusions:**

It is possible to detect a minor prevalence of precancerous and malignant lesions following post-operative procedures in POP. The assessment of the elderly through the use of risk-based evaluation merits attention for the purpose of early identification. This study offers valuable insights that can be utilized in preoperative counseling and enhancing the preoperative evaluation process.

## Introduction

Pelvic organ prolapse (POP) is a prevalent issue among women of the elderly [1], the prevalence being more than 50% in women aged over age 50 years [2]. Vaginal hysterectomy is a common means of treating apical compartment prolapses. Appropriate preoperative evaluation should be undertaken prior to performing this procedure. The preoperative evaluations include preoperative blood tests, cervical cancer screening, ultrasonography and endometrial sampling in patients with abnormal uterine bleeding. According to data currently available, the reported rate of missing to detect malignant or premalignant gynecologic pathology in women with uterine prolapse following preoperative evaluation is 0.3% and 0.8% [3,4] and 2.6% and 5.7% [5,6], respectively. The prevailing global inclination in the management of POP is shifting towards uterine-preserving surgical interventions. A subset of patients expresses a preference for preserving the uterus, even in cases where uterine prolapse is present. Consequently, uterine-preserving procedures such as sacrospinous hysteropexy, high uterosacral ligament suspension and sacrohysteropexy have gained popularity, particularly in Western nations [7,8]. Therefore, it is imperative to investigate the possibilities of detecting unforeseen pathological conditions following hysterectomy for POP, even among patients considered to have a low risk profile [9].

The objective of this study was to evaluate the prevalence of precancerous gynecological lesions and gynecological cancer among patients who did not have relevant symptoms and had normal preoperative assessments. These patients were scheduled to have vaginal hysterectomy for POP. The results of our study have the potential to significantly contribute to the preoperative assessment and counseling process, especially in relation to the identification of precancerous gynecological lesions and gynecological cancer in patients who may appear to have a low risk.

## Methods

This retrospective cohort study was conducted in Ramathibodi Hospital, Mahidol University, Bangkok, Thailand, a tertiary care center and university hospital. The study protocol was approved by the Committee on Human Rights Related to Research Involving Human Subjects, Faculty of Medicine, Ramathibodi Hospital, Mahidol University, which is based on the Declaration of Helsinki. (ID 12-61-21).

The study cohort comprised all patients who had been diagnosed with POP and undergone vaginal hysterectomy from January 2009 to September 2018 in our institution. Patients with previously diagnosed precancerous gynecological lesions or gynecological cancer, abnormal preoperative findings, abnormal cervical cancer screening, abnormal endometrial biopsies, or incomplete operative notes or pathological results were excluded. The electronic medical records of eligible patients were reviewed to collect relevant patient characteristics and the final histopathological diagnosis of the hysterectomy specimen. Patient characteristics collected include age, body mass index (BMI), parity, menopausal status, duration of menopause, hormonal use, underlying disease including hypertension, diabetes mellitus, dyslipidemia, sexually transmitted diseases, breast cancer, heart disease, thyroid disease, osteoarthritis of the knee and any diseases reported by patients in their medical records, presenting symptoms, history of abnormal vaginal bleeding, stage of POP and procedure performed. The classification of POP stages has been established as a standard recommendation in the medical literature, based on the consensus reached by the International Urogynecology Association (IUGA) [10]. These stages are determined through the utilization of POP-Q measurements and are categorized into a total of four stages. Stage III and stage IV of POP were defined as advanced stages.

The preoperative evaluation included a cervical cancer screening done within 1 year preoperatively in all patients. Asymptomatic patients were not routinely examined by preoperative trans-vaginal ultrasonography (TVU) or endometrial sampling (ES), whereas patients with abnormal uterine bleeding such as menometrorrhagia or postmenopausal bleeding were evaluated by TVU and ES regardless of endometrial thickness. Postmenopausal bleeding was defined as bleeding occurring more than 1 year after menopause. The ovaries were evaluated only by pelvic examination because, as mentioned above, preoperative TVU was not routinely performed. In this study, precancerous gynecological lesions and gynecological cancer of the uterus and cervix were assessed. According to the Endometrial Collaborative Group (2000) and World Health Organization 2014 classification of endometrial hyperplasias [11], uterine precancerous lesions include atypical endometrial hyperplasia or endometrial intraepithelial neoplasia. According to the American Society for Colposcopy and Cervical Pathology (ASCCP, 2012) [12], precancerous cervical lesions include high-grade intraepithelial lesions, which include cervical intraepithelial neoplasia (CIN) II and CIN III.

All statistical analyses were performed using STATA V.16.0. Qualitative data are presented as percentage and continuous data as mean ± SD. Associations between primary outcome and variables were calculated with Student’s *t*-test, Mann–Whitney *U* test and Fisher’s exact test. *p* Values less than 0.05 were considered to denote statistical significance.

## Results

The electronic medical records of 533 patients were reviewed and three patients excluded because their preoperative Pap test results were abnormal, leaving 530 patients in this study. Their mean age was 66.8 years and 97.36% of them were postmenopausal. The mean BMI was 25.2 kg/m^2^. The most common presenting symptom was a mass protruding from vagina. Advanced stage POP was diagnosed in 88% of study patients. Relevant characteristics are shown in Table 1.

**Table 1.** Clinical and gynecological characteristics of patients undergoing vaginal hysterectomy for pelvic organ prolapse.

Variables	*n* (%)
Age (years)^a^	66.8 (7.98)
Body mass index (BMI) (kg/m2)^a^	25.2 (4.05)
Parity^a^	3.1 (1.71)
Vaginal delivery^a^	3.1 (1.71)
Postmenopausal	516 (97.36)
Duration of postmenopause^a^	15.4 (8.03)
Hormonal treatment	0 (0)
*Comorbidity*	
Hypertension	321 (60.6)
Diabetes mellitus	124 (23.4)
Dyslipidemia	165 (31.1)
Sexually transmitted disease	4 (0.8)
Breast cancer	9 (1.7)
Heart disease	9 (1.7)
Thyroid disease	18 (3.4)
Osteoarthritis of the knee	15 (2.8)
*Stage of pelvic organ prolapse*	
Stage I	0 (0)
Stage II	63 (11.9)
Stage III	290 (54.7)
Stage IV	177 (33.4)
*Presenting symptoms*	
Mass protruding per vagina	520 (98.1)
Stress urinary incontinence	44 (8.3)
Urgency urinary incontinence	23 (4.3)
Mixed urinary incontinence	28 (5.3)
Urinary difficulty	28 (5.3)
Urinary tract infection	3 (0.57)
Postmenopausal bleeding	15 (2.8)
Duration of symptoms (months)^b^	12 (1–240)
Length of hospital stay (days)^a^	3.4 (1.3)

^a^ Mean (SD).

^b^ Median (range).

All patients had undergone vaginal hysterectomy. Some other procedures were performed concomitantly to address individual patient’s requirements. Anterior colporrhaphy was the most common concomitant procedure, being performed in 445 patients (84%), followed by posterior colporrhaphy in 316 patients (59.6%) (Table 2).

**Table 2.** Procedures performed in patients undergoing vaginal hysterectomy for pelvic organ prolapse.

Procedure	*n* (%)
Vaginal hysterectomy	530 (100)
*Colporrhaphy*	
Anterior	445 (84.0)
Posterior	316 (59.6)
Sacrospinous ligament fixation	30 (5.7)
McCall culdoplasty	22 (4.2)
Cystoscopy	27 (5.1)
*Salpingo-oophorectomy*	
Unilateral	11 (2.1)
Bilateral	5 (1.0)

Fifteen of the 530 patients (2.84%) presented with postmenopausal bleeding and were evaluated with TVU and ES. The pathological findings were benign in all cases.

Nine of the 530 patients (1.7%) were found to have precancerous gynecological lesions or gynecological cancer on pathological examination of their operative specimens. Atypical endometrial hyperplasia was found in one patient (0.19%). Three patients (0.57%) were diagnosed with endometrial carcinoma; all three were postmenopausal (Table 3). Their pathological diagnoses were endometrial cancer of endometrioid type, grade I. All three of these patients subsequently underwent complete surgical staging, whereas the patient with atypical endometrial hyperplasia received no further treatment. These patients’ details are shown in Table S1. There were no cases of cervical cancer. Precancerous cervical lesions were found in five patients (0.95%): CIN II (0.38%) and CIN III (0.57%) (Table 3).

**Table 3.** Prevalence of precancerous lesions and gynecological cancer in patients undergoing vaginal hysterectomy for pelvic organ prolapse.

Prevalence	*n* (%)
Precancerous gynecological lesions	6 (1.13)
*Cervix*	
CIN II	2 (0.38)
CIN III	3 (0.57)
Endometrium atypical simple hyperplasia	1 (0.19)
Gynecological cancer	3 (0.57)
Cervical cancer	0 (0)
Endometrial cancer	3 (0.57)

We compared the clinical characteristics of patients with and without precancerous gynecological lesions or cancer. The only variable that differed significantly between these two groups was the duration of menopause (*p* = 0.02) (Table S2).

## Discussion

In the aging population, POP is a common gynecological condition that encompasses a range of symptoms, including vaginal symptoms, lower urinary tract symptoms, defecatory dysfunction and sexual dysfunction [13]. Gynecologists and urogynecologists specializing in the management of POP in women typically conduct thorough preoperative evaluations. However, despite these comprehensive assessments, various factors may contribute to unforeseen challenges, including the potential occurrence of premalignant or malignant outcomes. This study examined the presence of premalignant and malignant pathology in women who underwent vaginal hysterectomy for POP. The prevalence of precancerous uterine lesions and endometrial cancer in our study are comparable with those with POP undergoing hysterectomy found in previous studies worldwide. A retrospective study performed in the UK found the incidence of unexpected endometrial carcinoma to be 0.8% [4]. All women were postmenopausal without any symptoms suggestive of endometrial cancer. The authors suggested that preoperative ultrasound examinations should be performed in all cases, followed by endometrial sampling in women with thickened endometrium. Another study conducted in the USA reported that 0.21% (1/466) of postmenopausal women without vaginal bleeding were found by pathological examination of hysterectomy specimens to have endometrial cancer [6]. This group found five patients had endometrial hyperplasia and one endometrial carcinoma, which is consistent with our findings of a prevalence of 3/516 (0.58%) endometrial cancers in postmenopausal women without vaginal bleeding. In our study, 15 postmenopausal women with bleeding and negative ES results had no evidence of endometrial hyperplasia or cancer at the time of hysterectomy.

In a prospective study performed in Kuwait, no cases of premalignant endometrial lesions or cancer were detected by pathological examination of hysterectomy specimens from 80 women diagnosed with POP who had negative preoperative diagnostic work-ups, including vaginal swab, cervical cancer screening, TVU and ES [2]. Their results confirm the accuracy of TVU as a screening tool for determining endometrial thickness and determining whether subsequent ES is indicated. In our study, all endometrial cancers were found in asymptomatic women without postmenopausal bleeding; however, TVU and ES were not routinely performed. No cases of cervical cancer were detected in our study; this is consistent with the findings of previous studies [2,4–6]. However, precancerous cervical lesions (CIN II, CIN III) were found in some studies. Elbiaa et al. [2] found CIN II in 6.25% and CIN III in 2.5% of their study cohort, despite their patients having been screened by conventional Pap smear test preoperatively. They concluded that this reflects moderate sensitivity to detecting premalignant cervical changes in their laboratory. In our study, the prevalence of CIN II was 0.38% (2/530) and of CIN III was 0.57% (3/530), which is less than previously reported. All five of our patients with CIN II and CIN III were postmenopausal and their Pap tests were negative with no transformation zone (TZ). Because the dynamic point changes in response to menopause, the TZ is located deep within the endocervix in postmenopausal women [14]. According to the updated ASCCP guidelines [12], a meta-analysis has shown that negative cytology has good specificity and negative predictive value despite the absence of a TZ. Negative cytological results with an absent TZ are reportedly found in 10–20% of patients, especially older women [12]. The findings of CIN II and CIN III by pathological examination of hysterectomy specimens may represent false negative Pap tests. In Thailand, a national survey of false negative Pap tests over 5 years showed a false negative rate among Thai women of 13.8% [15]. In our study cohort, the prevalence of CIN II/III was 0.95%, which is lower than the overall national rate. We concluded that, in our hospital, cervical cancer screening is an appropriate tool for screening for cervical abnormalities.

According to the findings of this study and existing literature, it has been observed that despite doctors maintaining the standard protocol for preoperative evaluation, there is a possibility of missing premalignant or malignant pathology, particularly in elderly patients. POP is a frequently encountered gynecologic condition that primarily affects the elderly, who are at high risk for gynecologic malignancy. It is also associated with the potential development of premalignant and malignant complications. Based on the previous research, it is evident that the primary objective in the field of gynecological cancers is to prevent and diagnose precancerous lesions. This goal is crucial for enhancing patient outcomes, minimizing the financial burden associated with disease management and extending the duration of follow-up care [16]. Furthermore, in the current era, there is a growing trend towards the utilization of precision medicine for the purpose of enhancing the quality of patient care, particularly in the realm of early detection [17]. According to the authors’ perspective, it is advisable to conduct a comprehensive examination of patients’ medical histories, paying particular attention to abnormal symptoms as well as assessing the potential risk of malignancy. This approach may facilitate early detection of such conditions. Furthermore, a crucial aspect to consider is the occurrence of unexpected outcomes. It is imperative to engage in comprehensive and thorough counseling, specifically addressing potential premalignant or malignant pathology. Additionally, embracing a holistic approach is essential when considering potential future interventions and treatments for the case at hand.

The strengths of this study are the large number of patients we recruited, all of whom had complete records of operative notes and pathological findings. Still, our study had some limitations. It was a retrospective study and may have had information bias. Although our urogynecology clinic requires that all patients who are to undergo urogynecological operations undergo TVU evaluation at the first and the operative visit, not all patients undergo TVU prior to surgery. This may be because these patients were asymptomatic and had normal pelvic examinations. Our findings should increase awareness of the possibility of unexpected precancerous gynecological lesions and gynecological cancer.

In summary, this study provides data on the prevalence of precancerous gynecological lesions and gynecological cancer in patients undergoing vaginal hysterectomy for POP. These data can be used in counseling prior to both vaginal hysterectomy and uterine preservative POP surgery. We conclude that, even when all appropriate preoperative evaluations have been performed, precancerous gynecological lesions and cancer can still be encountered, especially in asymptomatic postmenopausal women. We suggest that TVU and ES should be routinely performed prior to POP surgery. Further studies on the cost-effectiveness of performing ES in every asymptomatic woman undergoing vaginal hysterectomy are recommended.

## References

[1] Drutz HP, Alarab M. Pelvic organ prolapse: demographics and future growth prospects. Int Urogynecol J. 2006;17(S1):1–5. doi:10.1007/s00192-006-0102-1. 16738741

[2] Elbiaa AA, Abdelazim IA, Farghali MM, et al. Unexpected premalignant gynecological lesions in women undergoing vaginal hysterectomy for utero-vaginal prolapse. Prz Menopauzalny. 2015;14(3):188–191. doi:10.5114/pm.2015.54344. 26528108 PMC4612556

[3] Grigoriadis T, Valla A, Zacharakis D, et al. Vaginal hysterectomy for uterovaginal prolapse: what is the incidence of concurrent gynecological malignancy? Int Urogynecol J. 2015;26(3):421–425. doi:10.1007/s00192-014-2516-5. 25293812

[4] Renganathan A, Edwards R, Duckett JR. Uterus conserving prolapse surgery—what is the chance of missing a malignancy? Int Urogynecol J. 2010;21(7):819–821. doi:10.1007/s00192-010-1101-9. 20135302

[5] Aydin S, Bakar RZ, Mammadzade A, et al. The incidence of concomitant precancerous lesions in cases who underwent hysterectomy for prolapse. J Clin Anal Med. 2016;7:676–680.

[6] Frick AC, Walters MD, Larkin KS, et al. Risk of unanticipated abnormal gynecologic pathology at the time of hysterectomy for uterovaginal prolapse. Am J Obstet Gynecol. 2010;202(5):e1-507–e4. doi:10.1016/j.ajog.2010.01.077. 20452498

[7] Ekici MA, Onal AC. Unexpected risk of gynecological malignant and premalignant disease in women undergoing hysterectomy for pelvic organ prolapse. Exp Biomed Res. 2020;3(1):56–62. doi:10.30714/j-ebr.2020157454.

[8] Pandey D, Inukollu PR, Shetty J, et al. Is it worth preserving the uterus? Unanticipated pathology in hysterectomy for pelvic organ prolapse (POP). Int J Reprod Contracept Obstet Gynecol. 2018;7(10):4145–4150. doi:10.18203/2320-1770.ijrcog20184143.

[9] Detollenaere RJ, DEN Boon J, Stekelenburg J, et al. Sacrospinous hysteropexy versus vaginal hysterectomy with suspension of the uterosacral ligaments in women with uterine prolapse stage 2 or higher: multicentre randomised non-inferiority trial. BMJ. 2015;351:h3717. doi:10.1136/bmj.h3717. 26206451 PMC4512203

[10] Haylen BT, Maher CF, Barber MD, et al. An International Urogynecological Association (IUGA)/International Continence Society (ICS) joint report on the terminology for female pelvic organ prolapse (POP). Int Urogynecol J. 2016;27(2):165–194. doi:10.1007/s00192-015-2932-1. 26755051

[11] Sobczuk K, Sobczuk A. New classification system of endometrial hyperplasia WHO 2014 and its clinical implications. Prz Menopauzalny. 2017;16(3):107–111. doi:10.5114/pm.2017.70589. 29507578 PMC5834925

[12] Massad LS, Einstein MH, Huh WK, et al. 2012 updated consensus guidelines for the management of abnormal cervical cancer screening tests and cancer precursors. J Low Genit Tract Dis. 2013;17(5 Suppl 1):S1–S27. doi:10.1097/LGT.0b013e318287d329. 23519301

[13] Aimjirakul K, Ng JJ, Saraluck A, et al. A retrospective cohort study on the prevalence, risk factors, and improvement of overactive bladder symptoms in women with pelvic organ prolapse. Int J Women Health. 2023;15:1039–1046. doi:10.2147/IJWH.S413670. PMC10352122 37469654

[14] Singer A. The uterine cervix from adolescence to the menopause. Br J Obstet Gynaecol. 1975;82(2):81–99. doi:10.1111/j.1471-0528.1975.tb02204.x. 1125147

[15] Koonmee S, Bychkov A, Shuangshoti S, et al. False-negative rate of papanicolaou testing: a national survey from the Thai society of cytology. Acta Cytol. 2017;61(6):434–440. doi:10.1159/000478770. 28738387

[16] D'augè TG, Giannini A, Bogani G, et al. Prevention, screening, treatment and follow-up of gynecological cancers: state of art and future perspectives. CEOG. 2023;50(8):160.

[17] Zhang PY, Yu Y. Precise personalized medicine in gynecology cancer and infertility. Front Cell Dev Biol. 2019;7:382. doi:10.3389/fcell.2019.00382. 32010694 PMC6978655

